# Maize Ribosome-Inactivating Protein Uses Lys158–Lys161 to Interact with Ribosomal Protein P2 and the Strength of Interaction Is Correlated to the Biological Activities

**DOI:** 10.1371/journal.pone.0049608

**Published:** 2012-12-12

**Authors:** Yuen-Ting Wong, Yiu-Ming Ng, Amanda Nga-Sze Mak, Kong-Hung Sze, Kam-Bo Wong, Pang-Chui Shaw

**Affiliations:** 1 Biochemistry Programme and Centre for Protein Science and Crystallography, School of Life Sciences, The Chinese University of Hong Kong, Shatin, New Territories, Hong Kong, China; 2 Department of Microbiology, The University of Hong Kong, Pokfulam, Hong Kong, China; Cleveland Clinic Lerner Research Institute, United States of America

## Abstract

Ribosome-inactivating proteins (RIPs) inactivate prokaryotic or eukaryotic ribosomes by removing a single adenine in the large ribosomal RNA. Here we show maize RIP (MOD), an atypical RIP with an internal inactivation loop, interacts with the ribosomal stalk protein P2 via Lys158–Lys161, which is located in the N-terminal domain and at the base of its internal loop. Due to subtle differences in the structure of maize RIP, hydrophobic interaction with the ‘FGLFD’ motif of P2 is not as evidenced in MOD-P2 interaction. As a result, interaction of P2 with MOD was weaker than those with trichosanthin and shiga toxin A as reflected by the dissociation constants (K_D_) of their interaction, which are 1037.50±65.75 µM, 611.70±28.13 µM and 194.84±9.47 µM respectively.

Despite MOD and TCS target at the same ribosomal protein P2, MOD was found 48 and 10 folds less potent than trichosanthin in ribosome depurination and cytotoxicity to 293T cells respectively, implicating the strength of interaction between RIPs and ribosomal proteins is important for the biological activity of RIPs. Our work illustrates the flexibility on the docking of RIPs on ribosomal proteins for targeting the sarcin-ricin loop and the importance of protein-protein interaction for ribosome-inactivating activity.

## Introduction

Ribosome-inactivating proteins (RIPs) comprise more than one hundred *N*-glycosidases [1] which cleave the *N*-glycosidic bond of adenine-2660 at 23S rRNA or adenine-4324 at 28S rRNA of ribosome specifically and irreversibly [2]–[3]. The corresponding adenine is situated at the α-sarcin ricin loop (SRL) which belongs to the GTPase centre of ribosome and actively involved in the interaction with the elongation factors [4]. Upon depurination of the SRL by RIPs, elongation factors fail to form a stable complex with ribosome and thus delivery of aminoacyl-tRNA to ribosome and translocation of peptide are prohibited, inevitably causing the cease of protein synthesis and cell death [5]–[7].

At present, about 30 RIPs have their structures solved and it is found that the structures and catalytic residues of RIPs are highly similar. Typically, the globular RIP is divided into a large N-terminal domain and a small C-terminal domain, creating an active cleft holding the five invariant catalytic residues [8]–[9]. Despite sharing highly conserved structure and catalytic centre, the potency and specificity of RIPs on ribosome vary [10]–[12].

Ricin has been demonstrated to depurinate both 23S and 28S rRNA isolated from the ribosome at the conserved SRL with similar k_m_ and k_cat_ values although it cannot inactivate the prokaryotic ribosome. In addition, the k_cat_ value of depurination using eukaryotic ribosome as substrate is over 80,000 folds higher than using naked 28S rRNA [13]. These indicate ribosomal proteins are involved in the biological activity of RIPs.

Among the studies aiming at finding the interacting ribosomal proteins, human P-proteins have been found in several occasions. Trichosanthin (TCS), shiga-like toxin 1 (SLT-1) and ricin A chain (RTA) have been shown to interact with the last eleven residues of human P-proteins [14]–[16]. Also, RTA has been crosslinked to human L10e [17] which is identical to the P-protein P0.

On the contrary, pokeweed antiviral protein (PAP) has been found to interact with yeast ribosomal protein L3 [18]–[19] and not require the C-terminus of P-proteins for its ribosome depurinating activity [20]. It has also been found that yeast L3 with Trp-255 and Pro-257 mutated is resistant to PAP while those deprived of P1 and P2 are less vulnerable to the deleterious effect of RTA and shiga toxin [18], [21]–[22].

To understand why RIPs exhibit different ribosome specificity despite their highly similar structure, several regions of major structural difference between PAP and RTA were swapped and tested. The swapping weakened but did not totally abolish the prokaryotic ribosome depurinating activity of PAP, suggesting the dissimilar regions alone are not responsible for the ribosome specificity [23]. Conversely, substituting Gly209-Lys225 in PAP with the corresponding region of karasurin-A greatly reduced the prokaryotic ribosome depurinating activity of PAP [24]. Alignment of amino acid sequences of PAP and karasurin-A has revealed a higher charge density in PAP at this region but whether the charges contribute to the prokaryotic ribosome specificity of PAP remains unknown.

Our group has previously solved the structure of TCS complexed with a 11 amino acid-peptide (C11-P) corresponding to the conserved C-terminus of P-proteins. This C11-P peptide could fit to the corresponding groove of RTA, STL-1 and saporin but not PAP, implying PAP may get access to the ribosome through a different route that does not require any P-protein [25].

Recently, we have also solved the crystal and solution structures of maize RIP [26]–[27]. Maize RIP is an atypical RIP that contains a 25-amino acid internal peptide in the precursor form (pro-RIP). The internal peptide is absent in most RIPs and represses the activity of maize RIP by at least 600 folds [28]–[29] and pro-RIP displays a lower affinity to rat liver ribosome in comparison to the active form [26]. We postulated the internal peptide creates a steric hindrance causing a crash between the protein and rRNA, thereby prohibiting the protein to inactivate the ribosome and reducing its activity [26]. By chemical shift perturbation NMR experiment, we have shown that amino acid residues at the N-terminal domain of MOD, the active form of maize RIP, are perturbed upon the addition of ribosomal protein P2.

In this report, we demonstrate that among all the positively charged residues on the surface of MOD, only Lys158-Lys161 take part in the interaction with P2. The P2-binding site of MOD is very different from other known RIP-P2 interaction. The affinity of MOD to P2 is weaker than those of TCS and shiga toxin A chain by 1.7 folds and 5.3 folds respectively and is likely due to the lack of hydrophobic interaction with P2. Additionally, MOD was found less potent than TCS in *N*-glycosidase activity and cytotoxicity by 48 and 10 folds respectively. Our findings illustrate RIPs can use various regions to dock on the same ribosomal target in response to the subtle differences in the structures and the affinity between RIPs and ribosome plays a role in the activity of the RIPs.

## Materials and Methods

### Cloning and site-directed mutagenesis of RIPs

Maize [Δ1–16, Δ287–300]-Pro-RIP (Pro-RIP-WT in short) and [Δ1–16, Δ163–164, Δ167–189, Δ287–300]-Pro-RIP (MOD-WT in short) were prepared as described [26]. MOD [K158A-K161A] was constructed by polymerase chain reaction (PCR) using mutagenic primers and subsequently cloned into pET3a (Novagen). For His-Myc-tagged MOD (Myc-MOD), nucleotides coding for the myc tag were inserted at the 3′ of the gene of MOD by PCR. The construct was then cloned into pET28a (Novagen). Trichosanthin (TCS) was cloned into pET3d (Novagen) as described [30]. His-SUMO-tagged shiga toxin A chain inactive mutant (Stx [E167A, R170A], StxA in short) was cloned into pRSETA (Invitrogen) pre-cloned with DNA encoding His-SUMO. C-terminal His-tagged MOD and TCS were cloned into pET28a while P2 and its variants P2 [ΔC5], P2 [ΔC10] were cloned into pET8c similarly as described [14].

### Protein expression and purification

Maize RIP variants, TCS and P2 variants were expressed and purified as described before [14], [26], [30]. His-myc tagged MOD and C-terminal His-tagged MOD and TCS were expressed in *E. coli* BL21(DE3)pLysS under the induction of 0.4 mM IPTG. The bacterial pellets were resuspended and sonicated in His buffer A (20 mM sodium phosphate buffer, 800 mM NaCl, 50 mM imidazole, pH 7.4), the soluble lysate was then subject to HisTrap High Performance column (GE Healthcare) for affinity purification. The proteins were then eluted using His buffer B (20 mM sodium phosphate buffer, 300 mM NaCl, 300 mM imidazole, pH 7.4).

His-SUMO-StxA was expressed in *E. coli* BL21(DE3)pLysS at 25°C for 5 hours under the induction of 0.4 mM IPTG and was firstly isolated as mentioned above. Eluate was then treated with His-SUMO-protease for removing the His-SUMO tag and dialysed against His buffer A at 4°C for 16 hours before loaded to HisTrap to recover the protein from the flow through.

### Purification of rat liver ribosome

Rat liver ribosome was prepared according to the protocol in Spedding [31] and a brief summary was given in Mak *et al.* [26]. The quality of isolated ribosome was checked by measuring its absorbance at 260 nm and 280 nm and a ratio of A_260_/A_280_ higher than 1.8 indicates a clean ribosome preparation was achieved.

### *In vitro* pull-down assay with ribosome

MOD, pro-RIP and MOD variants were diluted in coupling buffer (200 mM NaHCO_3_, 500 mM NaCl, pH 8.3) before immobilization. 150 µl suspension of NHS-activated Sepharose 4 Fast Flow (GE Healthcare) was rinsed with 10 bead-volumes of ice-cold 1 mM hydrochloric acid (HCl) and 2 mg MOD variant was added to the beads immediately after removal of HCl. After incubating for an hour, the protein solution was aspirated and unreacted succinimide ester on the beads was inactivated according to the instruction of the manufacturer. The beads were then washed with 1 ml elution buffer (20 mM sodium phosphate buffer, 1 M NaCl, pH 7.4) and equilibrated in phosphate buffer saline (PBS) for 15 minutes. 0.3 mg ribosome in 800 µl was then loaded to the beads for interaction with maize RIP variants for an hour. Following washing the beads extensively with PBS, the retained proteins were eluted using elution buffer and examined with SDS-PAGE.

### Crosslinking assay and western blotting

All RIPs and rat liver ribosome had their buffer exchanged to PBS using Amicon Ultra-15 prior to the crosslinking. Myc-MOD and a buffer control were labelled with 5-molar excess of crosslinker NHS-SS-Diazirine (SDAD, Pierce) for an hour. Then the unreacted succinimide ester of SDAD was quenched with 50 mM Tris and uncoupled SDAD was removed by Amicon Ultra-0.5. Labelled RIPs were then mixed with ribosomes at 90 µM and 0.8 µM respectively in PBS and allowed to interact at room temperature for 30 minutes in dark. Controls with either RIP or ribosome supplemented with SDAD-pre-treated buffer were set up at equal protein amount as stated above. The reaction mixtures were irradiated for an hour under a UV lamp (Spectroline) emitting light of 365 nm to activate the diazirine group to crosslink the interacting partners.

For MOD-P2 crosslinking, P2 was labelled by 2-molar excess of SDAD and incubated with MOD at equal concentration of 0.4 µM for 30 minutes and followed by an irradiation of an hour. Controls with MOD or P2 alone were set up as well.

After crosslinking, the samples were concentrated and subject to western blot of which the membrane was probed with monoclonal mouse anti-Myc (Cell Signaling Technology), in-house polyclonal rabbit anti-MOD and anti-P proteins (anti-P) antibodies and horseradish peroxidase conjugated goat anti-mouse (Bio-rad) and anti-rabbit (Invitrogen) antibodies.

### Surface plasmon resonance

BIAcore 3000 surface plasmon resonance biosensor (GE healthcare) was used for the kinetic study of RIP-P2 interaction. CM5 sensor chip was activated using the amine coupling kit (GE healthcare). In brief, P2 was diluted to 50 µg/ml with 10 mM sodium acetate, pH 3.8 and 40 µl of the sample was injected to the chip at a flow rate of 5 µl/min to obtain a final response unit (RU) of 1300. Ethanolamine was then injected to block the unreacted succinimide ester. A control flow cell was prepared with identical treatments but excluding P2 immobilization.

MOD, pro-RIP, MOD [K158A–K161A], TCS and StxA were dialyzed in the running buffer (20 mM Tris, 30 mM NaCl, 0.005% Tween 20, pH 7.4) and diluted to appropriate concentration for the kinetic analysis. Samples were injected at 30 µl/min for 3 to 5 minutes to attain equilibrium and running buffer was injected for 3 minutes to allow dissociation. The chip was then regenerated using 2 M NaCl. Triplicate was performed for every concentration of sample to obtain three independent RU. The RU values at equilibrium of every concentration were taken for fitting into the equation modified from the Michaelis-Menten equation by KaleidaGraph (Synergy software):
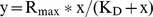
where Rmax is the maximum response unit and K_D_ is the dissociation constant.

### *In vitro* pull-down assay of RIPs with P2 and variants

2 mg of P2, P2 [ΔC5] or P2 [ΔC10] were immobilized to the NHS-activated Sepharose and MOD, pro-RIP, MOD [K158A–K161A], TCS or StxA was added at 55 µM in binding buffer (20 mM Tris, 30 mM NaCl, pH 7.4) for interaction. Elution was carried out using elution buffer as above.

Pull-down assay between RIPs and P2 was repeated using binding buffer of various ionic strengths. MOD, TCS or StxA was loaded to the beads in 20 mM Tris, pH 7.4 supplemented with 50 mM, 100 mM or 150 mM NaCl.

### N-glycosidase activity assay and quantitative PCR

50 µg rat liver ribosome was incubated with 5 nM MOD, pro-RIP, MOD [K158A-K161A] or TCS for 20 minutes at 30°C in the assay buffer A [20 mM Hepes (pH 7.6), 150 mM KCl, 10 mM MgCl_2_, 40 mM NH_4_Cl] at a final volume of 25 µl. 400 µl DEPC-treated water, 500 µl Trizol (Invitrogen) and 200 µl chloroform were added for phase separation of rRNA and proteins. After centrifugation, rRNA in the upper aqueous phase was precipitated with isopropanol and washed with 75% ethanol. The RNA pellet was then resuspended with DEPC-treated water and 500 ng of RNA was taken for cDNA synthesis using SuperScript II Reverse Transcriptase Kit (Invitrogen). The cDNA products were diluted for 10 folds for subsequent quantitative PCR (qPCR) using primers and protocol as described before [32]–[33] except 1 µl but not 4 µl of diluted cDNA was added. The relative *N*-glycosidase activity: Number of depurinated 28S rRNA at SRL/Number of total 28S rRNA, could be calculated from the threshold cycle (C_T_) values. The data were illustrated and statistically analyzed using unpaired t-test by Prism 5 (GraphPad Software).

Alternatively, 5 µg rRNA from rat liver ribosome was incubated with 15 µM MOD, pro-RIP, MOD [K158A-K161A] or TCS for 20 minutes at 55°C in assay buffer B [50 mM sodium citrate, pH 5.0] at a final volume of 10 µl. 2 µl reaction samples were taken for cDNA synthesis and 4 µl cDNA products were used as template in qPCR.

### Cytotoxicity to 293T cells

The cytotoxic effect of RIPs was compared using MTT (3-(4,5-Dimethylthiazol-2-yl)-2,5-diphenyltetrazolium bromide) cell proliferation assay. Human kidney embryonic 293T cells were cultured in Minimum Essential Medium supplemented with 10% fetal bovine serum (Invitrogen). 1×10^5^ 293T cells/well were seeded to a 96-well plate a night before the assay. Six different concentrations of RIPs were prepared in culture medium and added to the cells. After a 72-hour incubation, MTT was added at 0.5 mg/ml for incubation for 4 hours. 170 µl of medium was aspirated from the wells and replaced by the same volume of DMSO to dissolve the formazan crystal. OD_540_ was then measured for estimating the percentage of cell viability which was then plotted as a dose-response curve using Prism 5 (GraphPad Software). Individual EC_50_ value (the protein concentration that killed 50% of cells) was obtained from the dose-response curve and averaged EC_50_ values were computed from four independent trials each performed in triplicate.

### Cellular uptake of RIPs and western blotting

1×10^6^ of 293T cells were cultured in a culture dish (60×15 mm) overnight and 15 µM MOD or TCS fused with a C-terminal hexa-His tag was added to the cells for 6 hours. After washing off the untaken RIPs by PBS, cells were harvested and lysed in 200 µl PBS. Supernatant of the lysate was loaded for western blotting and probed with polyclonal rabbit anti-His (GenScript) and anti-rabbit antibodies. Beta-actin was also detected using monoclonal mouse anti-actin (GenScript) and anti-mouse antibodies for normalizing the loading amount of individual samples.

## Results

### All four residues of Lys158–Lys161 are involved in ribosome interaction

To examine whether ionic interaction is important for MOD-ribosome interaction, we have screened all the positively charged residues at the surface of MOD for their ability in binding ribosome.

**Figure 1:**
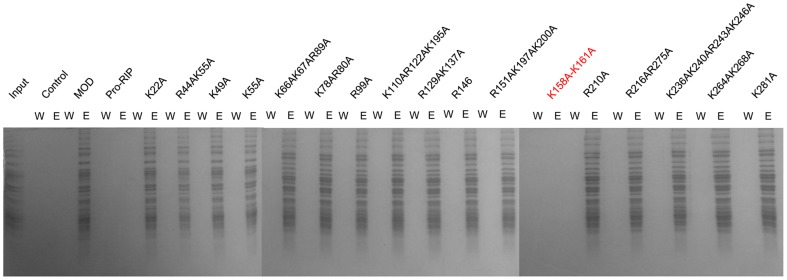
Screening of basic residues on MOD that are responsible for ribosome binding. The indicated residues were mutated to alanine and screened for their abilities to bind ribosome. W and E denote last wash and elution respectively.

A total of 17 variants were constructed with cluster of closely located basic residues mutated to alanine. Pull-down assay showed that all variants were able to interact with ribosome at similar extent as the wild type except MOD [K158A–K161A] (Figure 1), suggesting these four continuous lysine are responsible for the interaction with ribosome.

### MOD interacts with P2 on rat liver ribosome

By chemical perturbation experiment, we have shown that P2 may interact with the N-terminal domain of MOD [27]. Here, we examined if maize RIP really targets at P2 on the ribosome. SDAD-labelled Myc-tagged MOD was incubated with rat liver ribosome for interaction and permanent crosslinking was elicited by activation of the diazirine group of SDAD upon UV irradiation. Crosslinker SDAD (spacer-arm of 13.5 Å) was employed since it has two different chemical groups, N-hydroxysuccinimide ester and photoactivable diazirine, at two ends which allow a two-step crosslinking to avoid extensive self-crosslinking of ribosomal proteins that increases the complexity of western analysis.

Diazirine group of the crosslinker can react with any amino acid side chain or peptide backbone and thus inevitably generates a small amount of non-specific crosslinked bands which lead to undesired background on the western blot. Therefore, a C-terminal Myc-tagged variant of MOD was prepared for reducing the noise of the western blot, as the monoclonal antibody against the Myc-tag is more specific than the polyclonal anti-MOD antibody.

**Figure 2:**
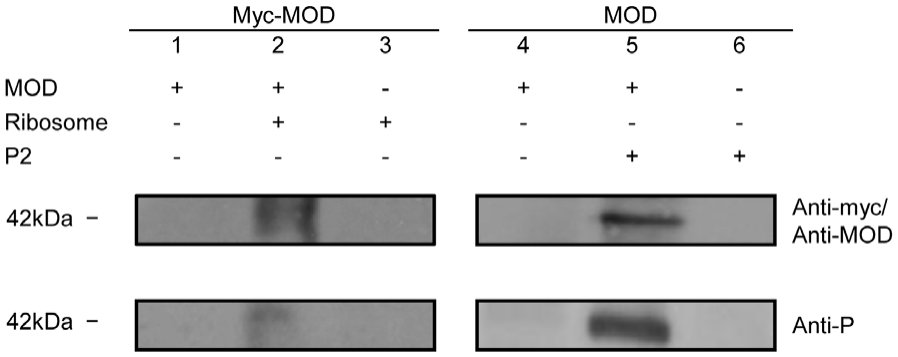
MOD can be crosslinked to rat liver ribosome and P2. Crosslinking reactions were carried with individual proteins (lanes 1 and 3, 4 and 6) or mixtures of two proteins (lanes 2 and 5) and subject for western analysis. Protein bands were detected by anti-myc, anti-MOD and anti-P antibodies.

The membranes were probed to detect MOD and P-proteins (Figure 2). In lane 2, a band of about 42 kDa could be detected on both blots, but absent in the negative controls (lanes 1 and 3). The size of the product coincided with the complex composed of one Myc-MOD and one P2 (42.3 kDa). Alternatively, crosslinking was also performed on mixture of MOD and P2 which yielded a band of slightly smaller size on both blots (MOD and P2 give a total size of 39 kDa), proposing MOD likely interacts with P2 when it encounters the ribosome.

As sizes of P-proteins P1 and P2 are similar (11.5 and 11.7 kDa), mass spectrometric analysis was employed to confirm whether the crosslinked partner is P2. Crosslinking was repeated on MOD immobilized to the resin and the crosslinked partner was collected by reducing the disulphide bond of the crosslinker and identified by mass spectrometry. The m/z spectrum of the product was found to match with that of P2 and thus MOD was crosslinked to P2 instead of P1 (data not shown).

### Lys158–Lys161 on Maize RIP are responsible for binding to P2

**Figure 3:**
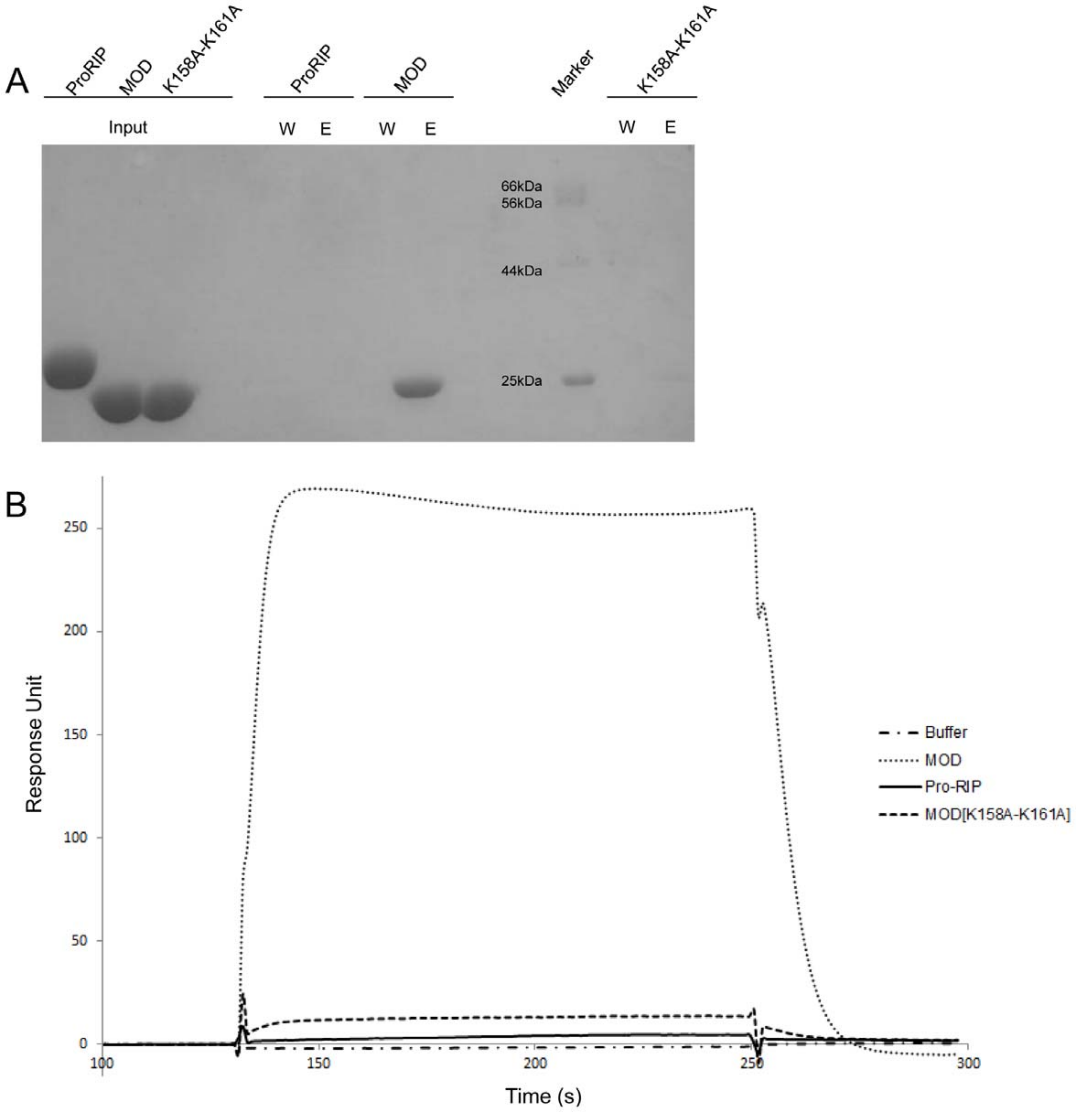
Lys158–Lys161 on MOD are responsible for the interaction between maize RIP and P2. **A) MOD does not bind to P2 when Lys158–Lys161 are mutated to alanine.** SDS-PAGE of the last wash (W) and elution (E) obtained from the pull-down assay of maize RIP and P2. Input indicates the purified proteins loaded to the column. **B) Sensorgram showing MOD but not pro-RIP and MOD [K158A-K161A] interact with sensor chip-immobilized P2.** 500 nM of maize RIP variants or running buffer were injected to the sensor chip for three minutes for association, followed by a dissociation using running buffer. Response unit (RU) was monitored in a real-time manner.

We next examined if maize RIP establishes a direct interaction with P2 via Lys158–Lys161. With same amount of P2 immobilized to the sepharose, identical amount of maize RIP variants were added for binding. The eluates were collected and analyzed by SDS-PAGE (Figure 3A) which showed that MOD but not pro-RIP could retain P2. The interaction between MOD [K158A–K161A] and P2 was highly diminished, implying that alanine substitution of these residues significantly impaired the interaction.

Surface plasmon resonance was employed to quantitatively compare the interaction between maize RIP and P2 (Figure 3B). MOD gave the highest response among the analytes tested with a K_D_ of 1037.50±65.75 µM while the response of pro-RIP and MOD [K158A–K161A] were negligible and comparable to the buffer control.

### Pro-RIP and MOD [K158A–K161A] were less potent in ribosome depurination and cytotoxicity to 293T cells

Next, we measured the relative depurinating activities of maize RIP and its variants on naked 28S rRNA and rat liver ribosome by estimating the relative amount of depurinated 28 rRNA and total 28S rRNA.

**Figure 4:**
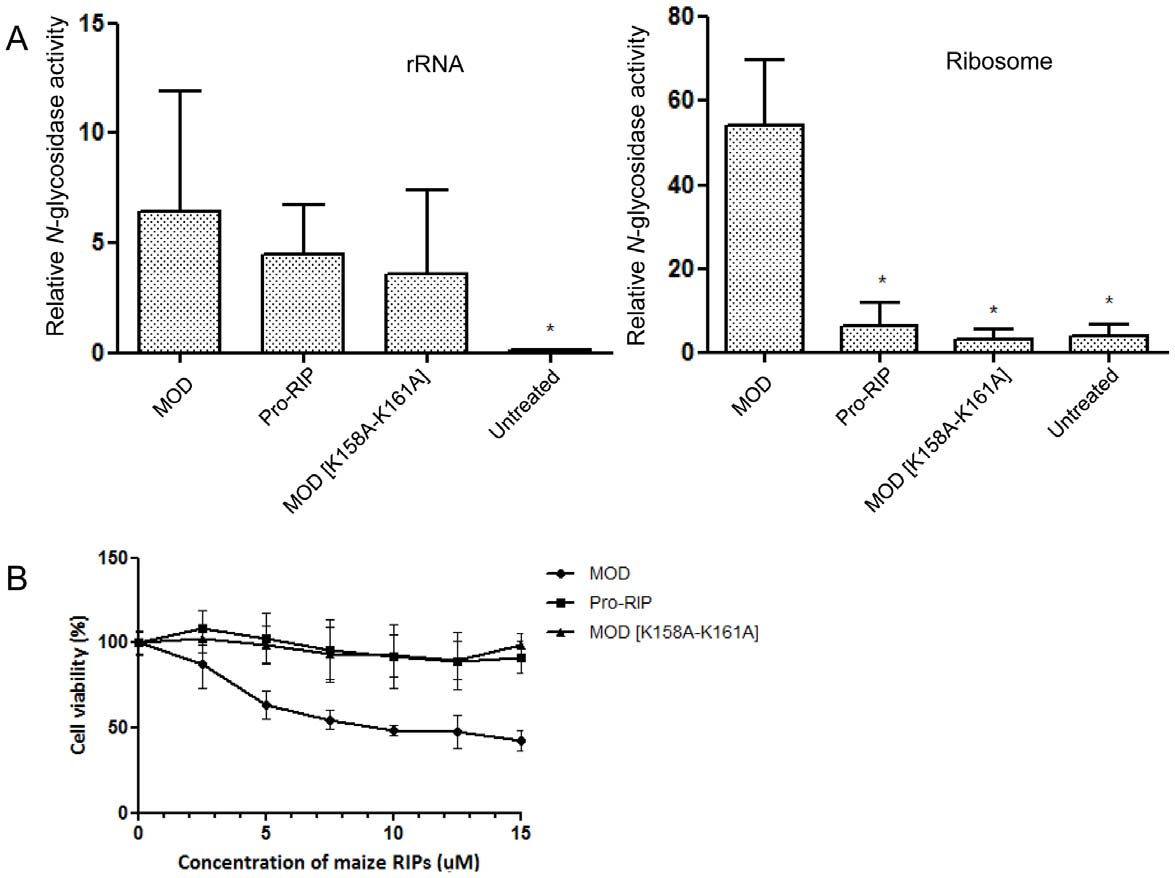
Relative *N*-glycosidase activity and cytotoxicity of MOD variants. **A) Relative** ***N*** **-glycosidase activities of MOD variants on isolated rRNA and rat liver ribosome.** The ratio was multiplied by an arbitrary factor for comparison. The data were expressed as mean ± S.D. from four independent trials each performed in duplicate. The *p* values were calculated using unpaired two-tailed Student's *t* test. * denotes a p-value<0.001 versus MOD. **B) Viability curves of 293T upon treatment of maize RIP variants.** Cell viability was determined by MTT cell proliferation assay. The data are expressed as the means ± S.D. and represent four independent trials each performed in triplicate.

It is observed that MOD, pro-RIP and MOD [K158A–K161A] cleaved naked 28S rRNA to a similar extent. Conversely, pro-RIP and MOD [K158A–K161A] negligibly depurinated 28S rRNA embedded in the ribosome (Figure 4A and Table 1). These suggested the three forms of maize RIP possess comparable catalytic activity and the difference in ribosome depurinating activity was due to their different affinity to P2. Consistently, pro-RIP and MOD [K158A–K161A] were much less toxic to 293T cells than MOD, as the former two variants did not show cytotoxicity up to 15 µM, while the IC_50_ of MOD was 7.88±1.57 µM (Figure 4B).

**Table 1:** Relative *N*-glycosidase activities of maize RIP and its variants on 28S rRNA and rat liver ribosome.

	rRNA		Ribosome	
RIPs	*N*-glycosidase activity (Mean ± S.D.)	Folds increased to untreated	*N*-glycosidase activity (Mean ± S.D.)	Folds increased to untreated
MOD	6.41±5.52	69.48	54.26±15.63	14.28
Pro-RIP	4.51±2.18	48.94	6.06±5.92	1.59
MOD [K158A-K161A]	3.58±3.85	38.85	3.24±2.28	0.85
Untreated	0.09±0.06	/	3.80±2.94	/

### Interaction of RIP-P2 under various ionic strengths

**Figure 5:**
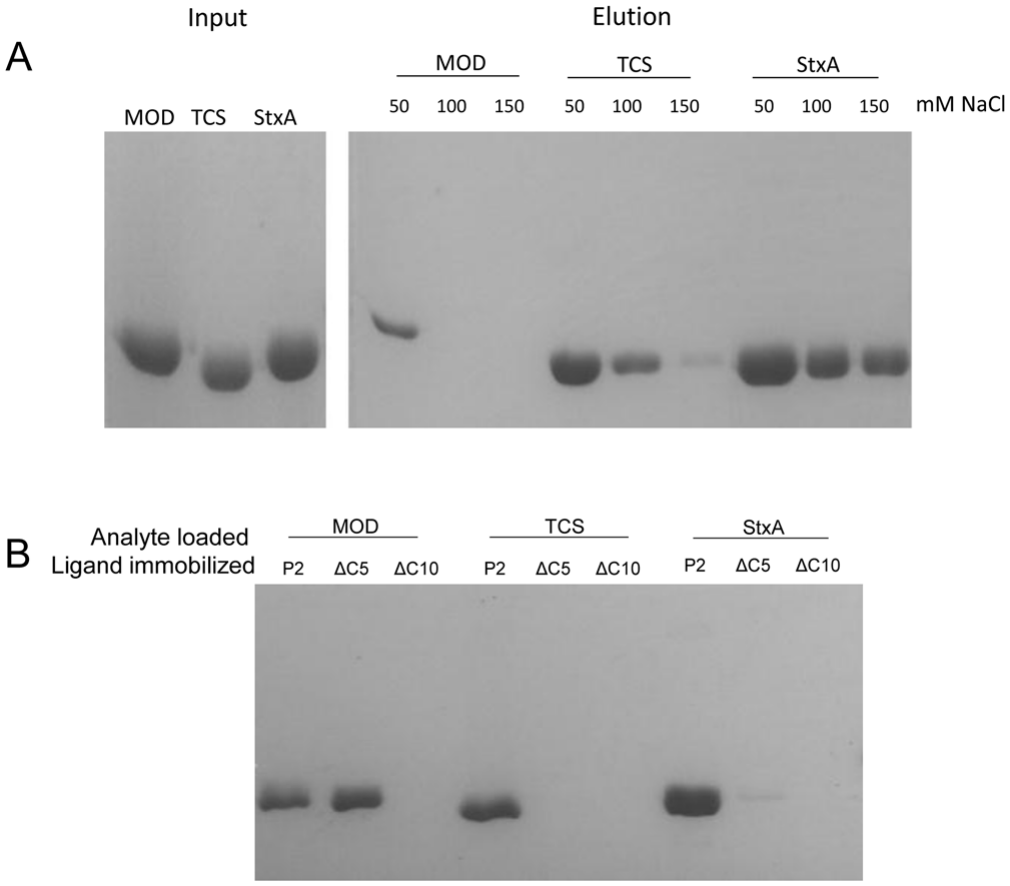
Interaction between RIPs and P2. **A) Interaction of MOD, TCS and StxA with P2 at different ionic strengths.** Pull-down assay was conducted on RIPs under various ionic strengths to compare their strength of interaction with P2. Input indicates the same amount of purified RIPs was loaded to the P2-sepharose column and proteins were eluted using buffer with 1M NaCl. **B) Interactions between RIPs and C-terminal truncated P2.** C-terminal truncated P2 variants were subject to pull down assay with RIPs. The C-terminal amino acid sequences of P2, P2 [ΔC5] and P2 [ΔC10] are: AEEKKDEKKEESEE**SDDDMGFGLFD** , AEEKKDEKKEESEE**SDDDMG** and AEEKKDEKKEESEE**S** respectively. The bold letters refer to the conserved residues in P-proteins.

Respecting MOD, TCS and StxA share the same partner P2, the next interest is to verify whether these RIPs interact with P2 with comparable affinity. Pull-down experiments on P2-column using buffers of different salt concentrations were carried out by loading equal amount of RIPs to the column. It was found that the amount of RIPs bound on P2-column was reduced with increasing salt, implying the interaction between RIP and P2 is highly electrostatic. MOD was least resistant to ionic challenge as it failed to bind to P2 at 100 mM or higher concentration of NaCl while TCS and StxA could still bind to P2 at 150 mM NaCl (Figure 5A).

### Maize RIP does not bind to the hydrophobic residues at the P2 tail

To examine the role of the highly consensus C-terminus of P-proteins for interacting with maize RIP and other RIPs, P2 variants lacking its last five or ten conserved residues were constructed for the pull-down assay.

It was observed that removing the last five residues ‘FGLFD’ of P2 heavily reduced the band intensities for TCS and StxA but imposed no effect on MOD-P2 interaction, implying MOD does not rely on the last five hydrophobic residues for interaction (Figure 5B). When the last ten residues of P2 were truncated, interactions of all tested pairs were abolished, indicating that the hydrophilic motif DDDMG on P2 is indispensable for RIPs to bind P2.

### MOD interacts with P2 at lower affinity than TCS and StxA

Surface plasmon resonance was carried out to quantify the strength of the interaction of each RIP-P2 pair. The K_D_ values of StxA-P2, TCS-P2 and MOD-P2 interaction were estimated to be 194.84±9.47 µM, 611.70±28.13 µM and 1037.50±65.75 µM respectively. The affinity between MOD and P2 is the weakest among three RIPs.

### MOD is less active than TCS

Regarding the binding site and affinity of interaction of MOD and TCS with P2 are very different, we would like to further test on their biological activities to verify if the activities are related to their affinities with P2.

The relative *N*-glycosidase activities on ribosome of MOD and TCS were estimated to be 54.26±15.63 and 2607.21±711.35 respectively, reflecting MOD was about 48 folds less potent than TCS in depurinating the ribosome. On the other hand, the relative *N*-glycosidase activities on naked rRNA of MOD and TCS were found to be 6.41±5.52 and 0.35±0.18 respectively. This reflected TCS was 18 folds less active on rRNA than MOD. The lower potency of MOD on ribosome is consistent to its lower affinity to P2.

The cytotoxicity to 293T cells also demonstrated a higher potency of TCS than MOD. For a fair comparison of the cytotoxicity, a hexa-His tag was incorporated into the C-termini of MOD and TCS so that equal amount of RIPs taken up by 293T could be ascertained by western blotting. The left panel of Figure S1 illustrates the same amount (20 ng) of RIPs was loaded. The right panel shows comparable intensity for MOD and TCS and hence a similar degree of cellular uptake of these proteins could be assumed. As determined from the viability curves, the effective protein concentration that killed 50% of cells (EC_50_) of MOD was 7.88±1.57 µM while that of TCS was 0.63±0.32 µM, implicating MOD was at least 10 folds less toxic to 293T than TCS.

## Discussion

RIPs interact with the SRL on the 28S rRNA and remove a specific adenine residue. It is believed RIPs target at specific ribosomal protein that is in proximity to their rRNA target SRL. Upon binding to RIPs, conformation of ribosomal proteins alters and subsequently increases the accessibility of the RIPs to SRL. Thus, ribosomal proteins can enhance the activity of RIPs by facilitating the latter to bind to the ribosome as well as to locate their target.

As proposed earlier, the interaction between RTA and ribosome can be characterized by a two-step binding model, consisting of a faster and stronger interaction involving particular ribosomal protein(s) and a slower and weaker interaction between the ribosomal surface and RIPs which may involves multiple binding sites on a single ribosome [34]. Ribosomal protein is believed to facilitate the depurinating activity of RIPs by guiding them to the SRL [14], [34]. Unable to bind to a target ribosomal protein(s) would inevitably compromise the interaction with ribosome as well as the activity of RIPs.

In the case of maize RIP, the P2-binding site on maize RIP was located at Lys158–Lys161, which is not at the C-terminus as in other RIPs, and their mutation severely compromised the interaction with ribosome. Our findings show the crucial role of electrostatic interaction in RIP-ribosome interaction and highlight the importance of interacting with specific ribosomal protein for RIPs to attain a good accessibility to ribosome.

**Figure 6:**
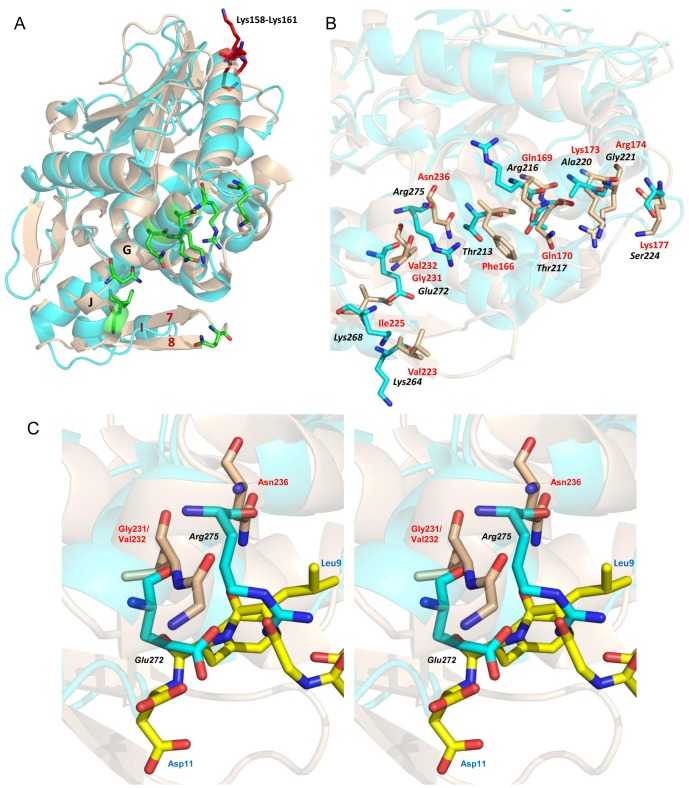
Residues on MOD that correspond to the C11-P interacting residues on TCS. **A) Superimposed structures of MOD (pdb: 2PQI) and TCS-C11-P complex (pdb: 2JDL).** The beta strands β7 and β8 in TCS (wheat) are replaced by the helix αI in MOD (cyan) while helices αG and αJ are conserved. P2-binding residues on MOD and TCS are distant apart as indicated in red and green respectively. **B) Comparison of C11-P interacting residues on TCS and the corresponding residues on MOD.** Structures of MOD and TCS are superimposed to locate the residues on MOD (colored in cyan and labelled in black and italic) corresponding to the C11-P interacting residues on TCS (colored in wheat and labelled in red). Many residues in these two RIPs are different, especially those at the hydrophobic patch of TCS (Phe-166, Val-223, Ile-225, Gly-231, Val-232 and Asn-236), suggesting MOD may interact with C11-P at a different site. **C) Stereo image zooming in the hydrophobic pocket of MOD (colored in cyan) with C11-P (colored in yellow).** The model reveals Arg-275 on MOD may crash with Leu-9 on C11-P while Glu-272 confronts directly Asp-11. Residues on TCS that interact with C11-P are highlighted in wheat for reference.

The interacting site on maize RIP for P2 was found to be different from other RIPs. Several pronounced structural differences were found between MOD and the typical topology of RIPs [26]. The P2-binding site on TCS is located at the interface of strands β7 and β8 and helices αG and αJ at which TCS establishes extensive hydrophilic and hydrophobic bonding with C11-P [25]. However, the antiparallel β7 and β8 are replaced by a short helix αI in MOD (Figure 6A). It has also been found that StxA and C11-P interact through hydrophilic and hydrophobic interaction [16]. We superimposed the structures of MOD and TCS-C11-P complex and found that the hydrophilic region Gln-170, Lys-173, Arg-174 and Lys-177 in TCS for interacting with C11-P are substituted by aliphatic or polar Thr-217, Ala-220, Gly-221 and Ser-224 in MOD that appear less favorable for the electrostatic interaction with the DDD motif of C11-P (Figure 6B).

Alternatively, the hydrophobic and slightly polar patch on TCS is replaced by a charged and highly polar surface on MOD. Phe-166, Val-223, Ile-225, Gly-231/Val-232 and Asn-236 in TCS are replaced by Thr-213, Lys-264, Lys-268, Glu-272 and Arg-275 in which the housing of hydrophobic FGLFD motif of C11-P is highly discouraged. Particularly, we noticed Arg-275 of MOD may crash with Leu-9 of C11-P while Glu-272 is facing the Asp-11 of C11-P (Figure 6C).

While MOD, TCS and StxA commonly rely on the tail of P2 for interaction, we observed that MOD docks at the DDDMG motif of P2 without the involvement of the FGLFD motif. This explains why the interaction between MOD and P2 is weaker and more salt-dependent. Examination of the hydrophobic amino acids around Lys158-Lys161 of MOD showed that they form a shallow cavity which may not be appropriate for the housing of the hydrophobic motif of C11-P. Alternatively, the positively charged Lys158–Lys161 in MOD are replaced by hydrophobic and polar residues Tyr-141, Tyr-142, Asn-143 and Ala-144 in TCS and less polar residues Ser-144, His-145, Ser-146 and Gly-147 in StxA which further supports MOD binds P2 very differently from those of TCS and StxA.

We also noticed that the DDDMG motif on P2 may contribute a more substantial role in the interaction for some RIPs. In the case of StxA, there was still some interaction with P2 with the deletion of the last five residues (Figure 5B).

Amino acid sequences of RIPs show that their ribosome binding motifs are highly variable and the ability to interact with the ribosomal stalk proteins is believed to attain independently through convergent evolution [35]. P-protein binding residues in TCS, StxA and MOD are not mutually conserved as seen from their superimposed structures and also not consensus in their respective homologous RIPs. Our findings further illustrate the flexibility on docking of RIPS on ribosomal proteins for the targeting of the sarcin-ricin loop.

Previous studies demonstrated RIPs could not elicit their full activity when the ribosome-binding residues were mutated or the ribosome is deficient in their ribosomal interacting partner [14], [18], [21], [22], [26], pointing out the importance of interaction with ribosomal proteins to the activity of RIPs. In the present study, we observed that the *N*-glycosidase activity and cytotoxicity of MOD were much lower than TCS, further ascertaining the role of ribosomal proteins for the enzymatic activity of RIPs and implying the strength of their interaction with P2 may play a role on the activity of RIPs.

In summary, we have shown that RIPs may use different regions to dock on the same ribosomal target depending on their structural features. In the case of maize RIP, it interacts with P2 at a novel binding site Lys158–Lys161 which only permits hydrophilic interaction with the DDDMG motif but not the hydrophobic tail of C11-P.

We have further illustrated that the strength of interaction between RIPs and ribosomal proteins is an important factor for the activity of RIPs. Lastly, our work provides an example on the use of protein-protein interaction but not the catalytic centre to modulate enzymatic activity and illustrates the flexibility on the docking of RIPs on ribosomal proteins for activity.

## Supporting Information

Figure S1 **Cellular uptakes of MOD and TCS are similar.** Same amount of RIPs was added to cells. Protein taken up by cells was detected by antibody against His-tag. Beta-actin was used as the loading control to ensure similar amount of cells was subject to the western analysis.
(TIF)

## References

[1] Girbes T, Ferreras JM, Arias FJ, Stirpe F (2004) Description, distribution, activity and phylogenetic relationship of ribosome-inactivating proteins in plants, fungi and bacteria. Mini Rev Med Chem 4: 461–76. 15180503 10.2174/1389557043403891

[2] Stirpe F (2004) Ribosome-inactivating proteins. Toxicon 44: 371–83. 15302521 10.1016/j.toxicon.2004.05.004

[3] Stirpe F, Battelli MG (2006) Ribosome-inactivating proteins: progress and problems. Cell Mol Life Sci 63: 1850–66. 16799768 10.1007/s00018-006-6078-7 PMC11136412

[4] Lancaster L, Lambert NJ, Maklan EJ, Horan LH, Noller HF (2008) The sarcin-ricin loop of 23S rRNA is essential for assembly of the functional core of the 50S ribosomal subunit. Rna 14: 1999–2012. 18755834 10.1261/rna.1202108 PMC2553751

[5] Montanaro L, Sperti S, Mattioli A, Testoni G, Stirpe F (1975) Inhibition by ricin of protein synthesis in vitro. Inhibition of the binding of elongation factor 2 and of adenosine diphosphate-ribosylated elongation factor 2 to ribosomes. Biochem J 146: 127–31. 167711 10.1042/bj1460127 PMC1165282

[6] Fernandez-Puentes C, Vazquez D (1977) Effects of some proteins that inactivate the eukaryotic ribosome. FEBS Lett 78: 143–6. 872934 10.1016/0014-5793(77)80292-5

[7] Brigotti M, Rambelli F, Zamboni M, Montanaro L, Sperti S (1989) Effect of alpha-sarcin and ribosome-inactivating proteins on the interaction of elongation factors with ribosomes. Biochem J 257: 723–7. 2930482 10.1042/bj2570723 PMC1135648

[8] Robertus JD, Monzingo AF (2004) The structure of ribosome inactivating proteins. Mini Rev Med Chem 4: 477–86. 15180504 10.2174/1389557043403837

[9] de Virgilio M, Lombardi A, Caliandro R, Fabbrini MS (2010) Ribosome-inactivating proteins: from plant defense to tumor attack. Toxins (Basel) 2: 2699–737. 22069572 10.3390/toxins2112699 PMC3153179

[10] Suh JK, Hovde CJ, Robertus JD (1998) Shiga toxin attacks bacterial ribosomes as effectively as eucaryotic ribosomes. Biochemistry 37: 9394–8. 9649321 10.1021/bi980424u

[11] Barbieri L, Ciani M, Girbes T, Liu WY, Van Damme EJ, et al (2004) Enzymatic activity of toxic and non-toxic type 2 ribosome-inactivating proteins. FEBS Lett 563: 219–22. 15063752 10.1016/S0014-5793(04)00286-8

[12] Narayanan S, Surendranath K, Bora N, Surolia A, Karande AA (2005) Ribosome inactivating proteins and apoptosis. FEBS Lett 579: 1324–31. 15733836 10.1016/j.febslet.2005.01.038

[13] Endo Y, Tsurugi K (1988) The RNA N-glycosidase activity of ricin A-chain. Nucleic Acids Symp Ser 139–42. 3226909

[14] Chan DS, Chu LO, Lee KM, Too PH, Ma KW, et al (2007) Interaction between trichosanthin, a ribosome-inactivating protein, and the ribosomal stalk protein P2 by chemical shift perturbation and mutagenesis analyses. Nucleic Acids Res 35: 1660–72. 17308345 10.1093/nar/gkm065 PMC1865052

[15] McCluskey AJ, Poon GM, Bolewska-Pedyczak E, Srikumar T, Jeram SM, et al (2008) The catalytic subunit of shiga-like toxin 1 interacts with ribosomal stalk proteins and is inhibited by their conserved C-terminal domain. J Mol Biol 378: 375–86. 18358491 10.1016/j.jmb.2008.02.014

[16] McCluskey AJ, Bolewska-Pedyczak E, Jarvik N, Chen G, Sidhu SS, et al (2012) Charged and hydrophobic surfaces on the A chain of shiga-like toxin 1 recognize the C-terminal domain of ribosomal stalk proteins. PLoS One 7: e31191. 22355345 10.1371/journal.pone.0031191 PMC3280276

[17] Vater CA, Bartle LM, Leszyk JD, Lambert JM, Goldmacher VS (1995) Ricin A chain can be chemically cross-linked to the mammalian ribosomal proteins L9 and L10e. J Biol Chem 270: 12933–40. 7759553 10.1074/jbc.270.21.12933

[18] Hudak KA, Dinman JD, Tumer NE (1999) Pokeweed antiviral protein accesses ribosomes by binding to L3. J Biol Chem 274: 3859–64. 9920941 10.1074/jbc.274.6.3859

[19] Rajamohan F, Ozer Z, Mao C, Uckun FM (2001) Active center cleft residues of pokeweed antiviral protein mediate its high-affinity binding to the ribosomal protein L3. Biochemistry 40: 9104–14. 11478877 10.1021/bi002851p

[20] Ayub MJ, Smulski CR, Ma KW, Levin MJ, Shaw PC, et al (2008) The C-terminal end of P proteins mediates ribosome inactivation by trichosanthin but does not affect the pokeweed antiviral protein activity. Biochem Biophys Res Commun 369: 314–9. 18282466 10.1016/j.bbrc.2008.01.170

[21] Chiou JC, Li XP, Remacha M, Ballesta JP, Tumer NE (2008) The ribosomal stalk is required for ribosome binding, depurination of the rRNA and cytotoxicity of ricin A chain in Saccharomyces cerevisiae. Mol Microbiol 70: 1441–52. 19019145 10.1111/j.1365-2958.2008.06492.x PMC2637795

[22] Chiou JC, Li XP, Remacha M, Ballesta JP, Tumer NE (2011) Shiga toxin 1 is more dependent on the P proteins of the ribosomal stalk for depurination activity than Shiga toxin 2. Int J Biochem Cell Biol 43: 1792–801. 21907821 10.1016/j.biocel.2011.08.018 PMC3206194

[23] Chaddock JA, Monzingo AF, Robertus JD, Lord JM, Roberts LM (1996) Major structural differences between pokeweed antiviral protein and ricin A-chain do not account for their differing ribosome specificity. Eur J Biochem 235: 159–66. 8631323 10.1111/j.1432-1033.1996.00159.x

[24] Nagasawa Y, Fujii K, Yoshikawa T, Kobayashi Y, Kondo T (2008) Pokeweed antiviral protein region Gly209-Lys225 is critical for RNA N-glycosidase activity of the prokaryotic ribosome. Phytochemistry 69: 1653–60. 18377939 10.1016/j.phytochem.2008.02.012

[25] Too PH, Ma MK, Mak AN, Wong YT, Tung CK, et al (2009) The C-terminal fragment of the ribosomal P protein complexed to trichosanthin reveals the interaction between the ribosome-inactivating protein and the ribosome. Nucleic Acids Res 37: 602–10. 19073700 10.1093/nar/gkn922 PMC2632931

[26] Mak AN, Wong YT, An YJ, Cha SS, Sze KH, et al (2007) Structure-function study of maize ribosome-inactivating protein: implications for the internal inactivation region and the sole glutamate in the active site. Nucleic Acids Res 35: 6259–67. 17855394 10.1093/nar/gkm687 PMC2094058

[27] Yang Y, Mak AN, Shaw PC, Sze KH (2010) Solution structure of an active mutant of maize ribosome-inactivating protein (MOD) and its interaction with the ribosomal stalk protein P2. J Mol Biol 395: 897–907. 19900464 10.1016/j.jmb.2009.10.051

[28] Walsh TA, Morgan AE, Hey TD (1991) Characterization and molecular cloning of a proenzyme form of a ribosome-inactivating protein from maize. Novel mechanism of proenzyme activation by proteolytic removal of a 2.8-kilodalton internal peptide segment. J Biol Chem 266: 23422–7. 1744135

[29] Hey TD, Hartley M, Walsh TA (1995) Maize ribosome-inactivating protein (b-32). Homologs in related species, effects on maize ribosomes, and modulation of activity by pro-peptide deletions. Plant Physiol 107: 1323–32. 7770526 10.1104/pp.107.4.1323 PMC157267

[30] Chan SH, Hung FS, Chan DS, Shaw PC (2001) Trichosanthin interacts with acidic ribosomal proteins P0 and P1 and mitotic checkpoint protein MAD2B. Eur J Biochem 268: 2107–12. 11277934 10.1046/j.1432-1327.2001.02091.x

[31] Spedding G (1990) Ribosomes and Protein Synthesis. Oxford: Oxford University Press. 9–12 p.

[32] Melchior WB Jr, Tolleson WH (2010) A functional quantitative polymerase chain reaction assay for ricin, Shiga toxin, and related ribosome-inactivating proteins. Anal Biochem 396: 204–11. 19766090 10.1016/j.ab.2009.09.024

[33] Law SK, Wang RR, Mak AN, Wong KB, Zheng YT, et al (2010) A switch-on mechanism to activate maize ribosome-inactivating protein for targeting HIV-infected cells. Nucleic Acids Res 38: 6803–12. 20558598 10.1093/nar/gkq551 PMC2965250

[34] Li XP, Chiou JC, Remacha M, Ballesta JP, Tumer NE (2009) A two-step binding model proposed for the electrostatic interactions of ricin a chain with ribosomes. Biochemistry 48: 3853–63. 19292477 10.1021/bi802371h PMC2677637

[35] Lapadula WJ, Sanchez-Puerta MV, Ayub MJ (2012) Convergent evolution led ribosome inactivating proteins to interact with ribosomal stalk. Toxicon 59: 427–32. 22245625 10.1016/j.toxicon.2011.12.014

