# Supplementary figures and images for: Maize Ribosome-Inactivating Protein Uses Lys158–Lys161 to Interact with Ribosomal Protein P2 and the Strength of Interaction Is Correlated to the Biological Activities
Source: PLoS One. 2012 Dec 12;7(12):e49608. doi: 10.1371/journal.pone.0049608 (PMC3520970; doi:10.1371/journal.pone.0049608)

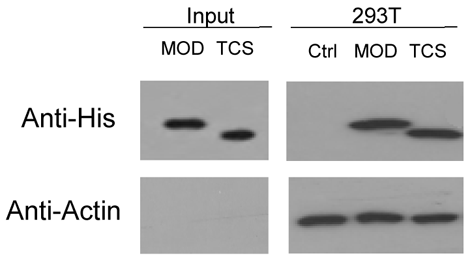

Supplement: Figure S1 — Cellular uptakes of MOD and TCS are similar. Same amount of RIPs was added to cells. Protein taken up by cells was detected by antibody against His-tag. Beta-actin was used as the loading control to ensure similar amount of cells was subject to the western analysis. (TIF) [file pone.0049608.s001.tif]
